# Genetics of Diffuse Idiopathic Skeletal Hyperostosis and Ossification of the Spinal Ligaments

**DOI:** 10.1007/s11914-023-00814-6

**Published:** 2023-08-02

**Authors:** Hajime Kato, Demetrios T. Braddock, Nobuaki Ito

**Affiliations:** 1 Division of Nephrology and Endocrinology, The University of Tokyo Hospital, 7-3-1 Hongo, Bunkyo-Ku, Tokyo, 113-8655 Japan; 2 Osteoporosis Center, The University of Tokyo Hospital, Tokyo, Japan; 3 Department of Pathology, Yale University, New Haven, CT USA

**Keywords:** Ossification of the posterior longitudinal ligament, Diffuse idiopathic skeletal hyperostosis, Fibrodysplasia ossificans progressiva, Pyrophosphatase, FGF23, ENPP1, SCVR1

## Abstract

**Purpose of Review:**

The study aims to provide updated information on the genetic factors associated with the diagnoses ‘Diffuse Idiopathic Skeletal Hyperostosis’ (DISH), ‘Ossification of the Posterior Longitudinal Ligament’ (OPLL), and in patients with spinal ligament ossification.

**Recent Findings:**

Recent studies have advanced our knowledge of genetic factors associated with DISH, OPLL, and other spinal ossification (ossification of the anterior longitudinal ligament [OALL] and the yellow ligament [OYL]). Several case studies of individuals afflicted with monogenic disorders, such as X-linked hypophosphatemia (XLH), demonstrate the strong association of fibroblast growth factor 23-related hypophosphatemia with OPLL, suggesting that pathogenic variants in *PHEX*, *ENPP1*, and *DMP1* are associated with FGF23-phosphate wasting phenotype and strong genetic factors placing patients at risk for OPLL. Moreover, emerging evidence demonstrates that heterozygous and compound heterozygous *ENPP1* pathogenic variants inducing ‘Autosomal Recessive Hypophosphatemic Rickets Type 2’ (ARHR2) also place patients at risk for DISH and OPLL, possibly due to the loss of inhibitory plasma pyrophosphate (PP_i_) which suppresses ectopic calcification and enthesis mineralization.

**Summary:**

Our findings emphasize the importance of genetic and plasma biomarker screening in the clinical evaluation of DISH and OPLL patients, with plasma PP_i_ constituting an important new biomarker for the identification of DISH and OPLL patients whose disease course may be responsive to ENPP1 enzyme therapy, now in clinical trials for rare calcification disorders.

## Introduction

Spinal ligaments, including the anterior and posterior longitudinal spinal ligaments, and the yellow ligament, are essential physiologic tethers which knit together vertebrae to provide the rigidity and flexibility necessary to stiffen an axial spinal column bearing the mechanical loads of bipedal locomotion. Ossifications of the anterior longitudinal ligament (OALL), posterior longitudinal ligament (OPLL), and yellow ligament (OYL) are pathological conditions in which spinal ligaments calcify, unbalancing both upright posture and locomotion, and inducing neurological pain (called myelopathy) due to spinal canal stenosis. Myelopathy most frequently occurs in either OPLL and OYL, due to the location of the spinal cord between the posterior longitudinal ligament and yellow ligament. The prevalence of OPLL is reported to be significantly higher in East Asian populations (0.4–3.0%) than in other ethnicities (0.01–2.0%) [1–3]. OYL was most prevalent in East Asian populations (16.9–63.9%) [4–6], with the prevalence in Europe and USA limited to case reports [7–14]. Many studies have attempted to identify underlying risk factors of OPLL, and multiple genetic and environmental factors have been suggested as disease-causing or disease-modifying agents, but there is little understanding of factors responsible for the heterotopic ossifications in OPLL and DISH, and few effective therapies preventing the progression of the diseases.

Diffuse idiopathic skeletal hyperostosis (DISH) is a specific medical entity which also exhibits ectopic ossifications of spinal ligaments. DISH was first described as ‘ankylosing hyperostosis of the spine’ in 1950 [15], and is characterized by ossification of the ligaments and entheses most commonly in the spine but also in peripheral joints (e.g., Achilles tendon). The diagnostic criteria of DISH first proposed by Resnick and Niwayama require the presence of calcification and ossification along the ventrolateral aspects (i.e., OALL and OPLL) of at least four contiguous vertebral bodies, with or without localized pointed excrescences at intervening vertebral body-disk junctions [16]. Subsequent studies found that patients with severe DISH also tended to exhibit age and sex unmatched osteoporosis of the spine [17–19]. DISH is more frequent in men and mostly occurs after the age of 40, with a prevalence of 2–4% in the males and females over 40 years of age. The prevalence of DISH rise to 10% or more in those over 70 years of age, and increases to 28% in those over 80 years old [20, 21]. Moreover, DISH often coexists with OPLL, suggesting that shared genetic and environmental risk factors may predispose the development of spinal ligament ossification in both conditions.

Here, we review the literature examining the genetic background of patients with spinal ligament ossifications, and the development of spinal osteoporosis in patients with OPLL and DISH. Our objective is to identify genetic risk factors for OPLL and DISH using insights from the following sources: monogenic and endocrine disorders which induce spinal ligament ossification, disease susceptibility studies assigning genetic risk factors for the development of spinal ligament ossifications, and pathologic variants of related genes (either monoallelic or biallelic) discovered in individuals with spinal ligament ossifications, paying special attention to factors found in patients presenting with the presumptive diagnosis of DISH or OPLL. We also obtained the allelic frequency of each genetic risk factors from Tohoku Medical Megabank Organization (ToMMo) or genome aggregation database (gnomAD). gnomAD is currently the largest collection of population variation from sequencing data of more than 195,000 individuals across the world (http://www.gnomad-sg.org/), while ToMMo provides a collection of Japanese population variation from sequencing data of 38,000 Japanese individuals (https://jmorp.megabank.tohoku.ac.jp/).

## Genetic Background of DISH/Spinal Ligament Ossification

### Monogenic Disorders Frequently Associated With Spinal Ligament Ossification

#### X-Linked Hypophosphatemia

X-linked hypophosphatemia (XLH: OMIM #307,800) is a genetic disease caused by inactivating mutations in the *phosphate regulating endopeptidase homolog, X-linked* (*PHEX*) gene, inducing a fibroblast growth factor-23 (FGF23) mediated hypophosphatemia. The prevalence of XLH is 1 in 20,000 births (0.005%), and is the most common cause of hypophosphatemic rickets [22], and spinal ligament ossification is recognized as one of the most common afflicting comorbidities in XLH patients (Table 1) [23–25]. Several recent studies involving relatively large numbers of patients identified OPLL in XLH patients, including a cohort study of 59 adult patients with XLH which found OPLL and OYL in five (71%) and four (57%) patients among seven who underwent radiological exam, respectively (Table 1) [26]. Another study, using patient-reported outcomes (PRO) data involving 232 adult patients with XLH, found that 44 patients, or 19%, exhibited spinal stenosis (Table 1) [27]. Finally, our laboratory performed spine computed tomography (CT) on a total of 25 adult patients with XLH, identifying spinal ligament ossification (OALL, OPLL, or OYL) in 20 patients (or 80%), and OPLL in eight patients (or 32%), which is a prevalence of at least 10 times greater than that of OPLL in the general Japanese population (2–4%) [3, 28••]. These observations support the notion that *PHEX* is a genetic risk factor for spinal ligament ossification (Table 1).

**Table 1:** Monogenic disorders accompanied with the spinal ligament ossification

Diseases	Responsible gene	OMIM	Study design	Country	*N*	Methods	Incidence	Reference
XLH	*PHEX*	307,800	Prospective	USA	38	X-rays	36% (men) 13% (women)	[23]
			Prospective	USA	39^a^	Not described	41%	[24]
			Prospective	Norway	52^b^	X-rays	63%	[25]
			Prospective	United Kingdom	59^c^	X-rays, CT, MRI	57% (OPLL) 71% (OYL)	[26]
			Prospective	USA	232	Self-reported (Internet-based survey)	19%	[27]
			Retrospective	Japan	25	CT	32% (OPLL) 72% (OYL)	[28••]
ARHR1	*DMP1*	241,520	Case report	Finland	2	X-rays, MRI	NA	[29]
			Case report	Canada	2	X-rays, MRI	NA	[30]
			Case report	Lebanon	2	X-rays	NA	[31]
ARHR2	*ENPP1*	613,312	Case report	Japan	1	CT	NA	[32, 33]
FOP	*ACVR1*	135,100	Case report^d^	United Kingdom	1	X-ray	NA	[34]
			Case report^d^	Australia	1	X-ray, CT	NA	[35]
			Case report^e^	USA	1	CT	NA	[36•]

*XLH*, X-linked hypophosphatemia; *ARHR1*, autosomal recessive hypophosphatemic rickets type 1; *ARHR2*, autosomal recessive hypophosphatemic rickets type 2; *FOP*, fibrodysplasia ossificans progressiva; *MRI*, magnetic resonance imaging; *CT*, computed tomography; *OPLL*, ossification of the posterior longitudinal ligament; *OYL*, ossification of the yellow ligament; *NA*, not applicable

^a^Twenty-nine patients underwent imaging studies of the spine

^b^Forty-six patients underwent imaging studies of the spine

^c^Seven patients underwent imaging studies of the spine

^d^This case presented typical phenotype of FOP

^e^This case harbored p.K400E mutation and presented only DISH without other phenotypes associated with FOP

#### Autosomal Recessive Hypophosphatemic Rickets Type 1

Autosomal recessive hypophosphatemic rickets type 1 (ARHR1: OMIM #241,520) is also a member of inherited FGF23-related hypophosphatemia. The causative gene for ARHR1, *dentin matrix protein 1* (*DMP1*), was discovered in 2006 in three families by Lorenz-Depiereux et al. and two families by Feng et al. [37, 38], and spinal ligament ossification was subsequently described in several patients with ARHR1. Case reports of two patients with ARHR1, 66-year-old woman and 78-year-old man, found spinal ligament ossification in the cervical, thoracic, and lumbar spine (Table 1) [29], as did another case report in a 36-year-old proband and two siblings [30]. In the latter study, the proband developed a severe form of OPLL, while his 26-year-old sibling showed a slight ossification in the anterior longitudinal ligament. Additionally, a final case report also found diffuse spinal ligament ossification, including OALL and OPLL, in 45- and 47-year-old patients with ARHR1 (Table 1) [31]. Therefore, ARHR1 is second monogenic disorder in patients with inherited FGF23-related hypophosphatemia that is associated with spinal ligament ossifications.

#### Autosomal Recessive Hypophosphatemic Rickets Type 2

Biallelic pathogenic variants of *Ectonucleotide pyrophosphatase/phosphodiesterase 1* (*ENPP1*) have been identified in patients with a third FGF-23 mediated hypophosphatasia called ‘Autosomal Recessive Hypophosphatemic Rickets type 2’ (ARHR2: OMIM #613,312). This is of interest because biallelic pathogenic *ENPP1* variants were previously identified in patients with ‘Generalized Arterial Calcification of Infancy’ (GACI). GACI may present as early as the 2nd trimester, and by 6 months of age 50% of the afflicted infants succumb to disease. If affected infants reach 6 months of age, they stabilize, but these patients almost invariably develop ARHR2, which is associated with OPLL in adult patients. ENPP1 is producing plasma inorganic pyrophosphate (PP_i_) production through enzyme catalysis. Because plasma PP_i_ is a potent inhibitor of ectopic calcification, especially in the medial layers of arterial walls where elastin fibers reside, decreased plasma PP_i_ levels may be an important pathogenic mechanism for the increased collagen fiber calcification seen in spinal ligament ossification of OPLL and DISH. ARHR2 is very rare and only one patient in our laboratory was found with an extremely severe case of OPLL (Table 1) [32, 33]. Although reports of OPLL affecting ARHR2 patients are scarce due to the rarity of ARHR2, tiptoe walking mouse (ttw), first recognized almost 40 years ago as a murine model of OPLL, was later found to possess biallelic ENPP1 loss of function mutations, genetically linking *ENPP1* to OPLL [39]. Prospective phenotyping of 20 survivors of GACI further supports an association between *ENPP1* and OPLL, in which eight patients with homozygous or compound heterozygous *ENPP1* pathogenic variants who developed ARHR2 after surviving GACI were found with enthesopathies in the Achilles tendons or elbow joints, including in relatively young patients, aged 26 and 25 years [40, 41•]. The appearance of enthesopathies in both young ARHR2 patients and ttw mice strongly supports *ENPP1* as a genetic risk factor for the development of spinal enthesopathies present in OPLL.

#### Fibrodysplasia Ossificans Progressiva

Fibrodysplasia ossificans progressiva (FOP: OMIM #135,100) is a rare genetic disease characterized by progressive heterotopic ossification of soft tissues including muscles, tendons, and ligaments. FOP is caused by heterozygous gain-of-function mutation (mainly p. R206H) in the *ACVR1* gene which induces altered BMP receptor trafficking resulting in enhanced BMP signaling and ectopic bone formation [42]. Clinical studies identified spinal ligament ossifications, such as OALL or OYL, in FOP patients (Table 1) [34, 35]. Moreover, a recent study discovered a monoallelic p.K400E variant of *ACVR1* in a DISH patient, which was confirmed to be pathogenic by functional analysis demonstrating increased BMP signaling through K400E *ACVR1* in response to osteogenic BMPs [36•, 43].

### Model Animal for DISH

Mice lacking *SLC29A1*, *a nucleotide transporter* involved in purine metabolism, exhibit a phenotype similar to DISH [44–46]. However, unlike *ENPP1*, spinal ligament ossification resulting from *SLC29A1* mutations have not been reported in human to date.

### Endocrine Disorders Frequently Accompanied With the Spinal Ligament Ossification

Some endocrine disorders such as acromegaly, hypoparathyroidism, pseudohypoparathyroidism, and familial hypocalciuric hypercalcemia (FHH) have been reported to be associated with DISH or spinal ligament ossification,. Acromegaly is a disorder caused by the development of growth hormone (GH)-producing pituitary adenoma, resulting in increased GH and insulin-like growth factor-1 (IGF-1). Spinal ligament ossification has been reported in some acromegaly patients [47, 48], while another study of 30 acromegaly patents found a high incidence of DISH (47%) [49]. However, a direct association between acromegaly and OPLL was never established due to patient selection bias. A recent study by Hoshino et al. from our group evaluated CT spinal data from ten consecutive patients with acromegaly, discovering spinal ligament ossification in five patients (50%) [50]. In this report, factors associated with spinal ligament ossification, such as age and body mass index (BMI), and clinical activity of acromegaly such as GH and IGF-1 were compared in patients with and without spinal ligament ossifications, revealing that BMI was the only significant factor differentiating the two groups. While the number of patients in this study was not sufficient to establish significance, the study suggested that acromegaly and mutations in genes inducing familial gigantism, such as *AIP*, *GPR101*, *GNAS*, *MEN1*, and *PRKAR1A*, may be risk factors for the development of spinal ligament ossifications [51].

There are several case reports associating patients with long-term untreated hypoparathyroidism, pseudohypoparathyroidism, or FHH with spinal ligament ossifications [52–62]. In addition to these case reports, a study of 17 patients with hypoparathyroidism reported that nine patients (53%) presented with spinal ligament ossifications [63]. These disorders, which affect calcium metabolism, may be another predisposing condition for spinal ligament ossifications.

### Disease Susceptibility Genes for Spinal Ligament Ossification

To date, numerous genetic studies of OPLL patients investigating disease susceptibility genes have revealed genetic risk factors for the development of OPLL (Table 2). We review some important genes for bone and cartilage formation from candidate gene association studies below.

**Table 2:** Genes and variants susceptible to spinal ligament ossifications

Gene	Study design	Country	*N*	Variant	MAF (gnomAD/ToMMo)	OR	Reference
(A) OPLL							
*ACE*	Candidate gene association study	Korea	95 (OPLL) 274 (control)	rs4646994	Not in database	2.20^a^	[64]
*AHSG*	Candidate gene association study	Japan	711 (OPLL) 896 (control)	rs2077119	0.35/0.54	-	[65]
*BID*	Candidate gene association study	Korea	157 (OPLL) 222 (control)	rs8190315	0.036/0.11	2.66	[66]
				rs2072392	0.040/0.11	2.66	
*BMP2*	Candidate gene association study	China	192 (OPLL) 304 (control)	rs3178250^b^	0.22/0.40	-	[67]
	Candidate gene association study	China	57 (OPLL) 135 (control)	rs2273073^c^	0.016/0.099	-	[68]
				rs1049007	0.71/0.81	-	
	Candidate gene association study	China	420 (OPLL) 560 (control)	rs1116867	0.11/0.15	-	[69]
				rs965291^d^	0.65/0.85	-	
	Candidate gene association study	China	420 (OPLL) 506 (control)	rs235768	0.72/0.81	-	[70]
				rs2273073	0.016/0.099	-	
*BMP4*	Candidate gene association study	China	179 (OPLL) 298 (control)	rs17563^b^	0.44/0.21	1.57	[71]
	Candidate gene association study	China	450 (OPLL) 550 (control)	rs76335800	0.0016/0.00017	1.68	[72]
				c.-7-203C > T	Not in database	1.58	
*BMP9*	Candidate gene association study	China	450 (OPLL) 550 (control)	rs75024165	0.0048/0.0018	1.74	[73]
				rs34379100	0.20/0.013	1.95	
*BMPR-1A*	Candidate gene association study	China	356 (OPLL) 617 (control)	rs11528010	0.40/0.71	Not described	[74]
*CCDC91*	GWAS	Japan	1130 (OPLL) 7135 (control)	rs1979679	0.25/0.64	1.30	[75•]
*COL6A1*	Genome-wide linkage study, Candidate gene association study	Japan	342 (OPLL) 298 (control)	rs2276254	0.57/0.73	Not described	[76]
				rs35796750	0.51/0.68	Not described	
				rs2236486	0.32/0.61	Not described	
	Candidate gene association study	China	90 (OPLL) 155 (control)	rs17551710	0.16/0.21	0.34	[77]
				rs35796750	0.51/0.68	0.53	
	Candidate gene association study	Korea	162 (OPLL) 159 (control)	rs17551710	0.16/0.21	Not described	[78]
				rs2236486	0.32/0.61	Not described	
	Candidate gene association study	China	100 (OPLL) 100 (control)	rs201153092	0.000033/0.00017	18.49	[79]
				rs13051496	0.15/0.066	5.76	
*COL11A2*	Sib-pair linkage study, candidate gene association study	Japan	137 (OPLL) 183 (control)	Promoter (− 182) A > C^e^	NA	Not described	[80]
				rs1799907	0.32/0.23	Not described	
				rs1799910	0.50/0.34	Not described	
				rs1799911	0.23/0.23	Not described	
	Candidate gene association study	Japan	195 (OPLL) 187 (control)	rs1417877815	0.000026/not in database	1.84	[81]
				rs1799907	0.32/0.23	1.99	
*COL17A1*	Candidate gene association study	China	28 (OPLL) 100 (control)	rs805698	0.85/0.84	7.72	[82]
				rs4918079	0.72/0.70	4.00	
*ENPP1*	Candidate gene association study	Japan	323 (OPLL) 332 (control)	rs397832689	0.40/0.35	Not described	[83]
	Candidate gene association study	Japan	180 (OPLL) 265 (control)	rs75272847	0.034/0.015	3.01	[84]
*ESR1*	Candidate gene association study	Japan	120 (OPLL) 306 (control)	*Xbal* variant	NA	Not described	[85]
	Candidate gene association study	Japan	711 (OPLL) 896 (control)	rs9340799	0.31/0.18	Not described	[65]
				rs2228480	0.18/0.15	Not described	
*ESR2*	Candidate gene association study	Korea	164 (OPLL) 219 (control)	rs1256049	0.066/0.29	2.41	[86]
*FGFR1*	Candidate gene association study	Korea	157 (OPLL) 222 (control)	rs13317	0.22/0.37	1.35^a^	[87]
*HAO1*	GWAS	Japan	1130 (OPLL) 7135 (control)	rs2423294	0.16/0.16	1.43	[75•]
*HLA*	Family-based study	Japan	33 families	NA	NA	Not described	[88]
	Genetic-linkage study	Japan	91 affected sib-pairs from 53 families	NA	NA	Not described	[80]
	Family-based study	Japan	24 families	NA	NA	Not described	[89]
*IFNG*	Candidate gene association study	Japan	135 (OPLL) 222 (control)	rs2430561	0.36/0.10	1.26^a^	[90]
				rs3138557 (CA_n_ repeat)	NA	0.69 (CA_13_) 1.52 (CA_15_)	
*IL1B*	Candidate gene association study	Japan	120 (OPLL) 306 (control)	*Aval* variant^d^	NA	Not described	[85]
*IL15RA*	Candidate gene association study	Korea	166 (OPLL) 230 (control)	rs2228059	0.55/0.44	1.58^a^	[91]
	Candidate gene association study	China	235 (OPLL) 250 (control)	rs2228059	0.55/0.44	1.63	[92]
*IL17RC*	Candidate gene association study	China	100 (OPLL) 100 (non-OPLL)	rs199772854	0.000059/not in database	6.32	[79]
				rs76999397	0.035/0.043	4.67	
				rs189013166	0.0037/0.015	8.29	
*MiR499*	Candidate gene association study	Korea	207 (OPLL) 200 (control)	rs3746444	0.19/0.18	4.3	[93]
*NLRP1*	Candidate gene association study	Korea	74 (OPLL) 26 (control)	rs121502220	Not in database	Not described	[94]
*RSPH9*^f^	GWAS	Japan	1130 (OPLL) 7135 (control)	rs927485	0.60/0.86	1.37	[75•]
*RSPO2*	GWAS	Japan	1130 (OPLL) 7135 (control)	rs374810	0.40/0.38	1.35	[75•]
*RUNX2*	Candidate gene association study	China	82 (OPLL) 118 (control)	rs1321075	0.84/0.80	0.84	[95]
				rs12333172	0.15/0.22	0.94	
	Candidate gene association study	China	80 (OPLL) 80 (control)	rs1406846	0.50/0.56	5.67	[96]
				rs967588	0.15/0.27	0.47	
				rs16873379	0.13/0.27	0.48	
*RXRB*	Candidate gene association study	Japan	134 (OPLL) 158 (control)	c.*140A > T^e^	NA	Not described	[97]
				c.*561_562insC^e^	NA	Not described	
*SSH2*	Candidate gene association study	Korea	74 (OPLL) 26 (control)	rs12150220	0.33/0.041	Infinite	[94]
*STK38L*^*f*^	GWAS	Japan	1130 (OPLL) 7135 (control)	rs11045000	0.17/0.45	1.32	[75•]
*TGFB1*	Candidate gene association study	Japan	46 (OPLL) 273 (control)	rs1800470	0.59/0.48	Not described	[98]
*TGFB3*	Candidate gene association study	Japan	711 (OPLL) 896 (control)	rs2268624	0.77/0.60	1.46	[65]
				rs2284792	0.65/0.55	Not described	
*TGFBR2*	Candidate gene association study	Korea	21 (OPLL) 42 (control)	rs11466512	0.26/0.34	2.81	[99]
				rs56105708	0.00068/0.0072	8.73	
*VDR*	Candidate gene association study	Japan	63 (OPLL) 126 (control)	*Fok1* variant	NA	Not described	[100]
*VKORC1*	Candidate gene association study	Korea	98 (OPLL) 200 (control)	rs9923231^d^	0.32/0.90	5.22	[101]
(B) DISH							
*COL6A1*	Candidate gene association study	Japan, Czech	97 (Japan, DISH) 298 (Japan, control) 96 (Czech, DISH) 96 (Czech, control)	rs35796750^g^	0.51/0.68	Not described	[102]
*FGF2*	Candidate gene association study	Korea	157 (OPLL)^h^ 222 (control)	rs1476217	0.42/0.50	NA	[87]
				rs3747676	0.43/0.46	NA	
*PPP2RD*	Candidate gene association study	Portugal	65 (DISH) 118 (control)	rs34473884	0.19/0.23	1.79	[103]

gnomAD is currently the largest collection of population variation from sequencing data of more than 195,000 individuals across the world (http://www.gnomad-sg.org/). ToMMo provides a collection of Japanese population variation from sequencing data of 38,000 Japanese individuals (https://jmorp.megabank.tohoku.ac.jp/). Variant information is provided in accordance with dbsnp database (https://www.ncbi.nlm.nih.gov/projects/SNP/snp_summary.cgi) or GRCh38

*DISH*, diffuse idiopathic spinal hyperostosis; *OPLL*, ossification of the posterior longitudinal ligament; *MAF*, minor allele frequency; *gnomAD*, genome aggregation database; *ToMMo*, Tohoku Medical Megabank Organization; *OR*, odds ratio; *NA*, not applicable; *GWAS*, genome-wide association study

^a^An odds ratio in codominant model

^b^A significant association was observed only in male patients

^c^This SNP was associated with the extent of OPLL

^d^A significant association was observed only in female patients

^e^Variant information was described as the original version because the allele at the reported location was different in GRCh38 or variant was not recorded in dbsnp database

^f^Located within 1 Mb of SNP significantly associated with OPLL

^g^A significant association was observed only in Japanese patients with DISH

^h^Three patients with DISH were included

*COL11A2* encodes collagen type XI alpha 2 chain, which is the main collagenous component of cartilage. Variants of this gene were associated with OPLL (promoter [− 182] A > C [the allele at promoter [− 182] is C in GRCh38]); therefore, the actual SNP introduced in this article is uncertain. The nearest reported SNP (A > C) around the promoter [− 182] is rs1799905 (promoter [− 186], rs1799907, rs1799910, rs1799911) in a candidate gene association study involving 137 Japanese cases and 183 controls [80]. Another candidate gene association study involving 195 Japanese cases and 187 controls found that a novel SNP in *COL11A2* (rs1417877815) along with the abovementioned SNP (rs1799907) was associated with OPLL [81]. Additionally, *COL6A1* was reportedly associated with OPLL in some candidate gene association studies (Table 2) [76–79].

One of the key regulators of bone metabolism, BMP2, is reportedly associated with OPLL. Several SNPs in *BMP2* (rs3178250, rs2273073, rs235768, rs1049007) or SNPs located upstream or downstream of *BMP2* (rs1116867, rs965291) are reported to be significantly associated with the occurrence or the severity of OPLL in several candidate gene association studies involving Chinese patients and control subjects [67–70]. Moreover, mutation analysis without control subjects using targeted next-generation sequencing revealed a deleterious coding variant of *BMP2* with rare allele frequencies among a total of 50 Chinese patients with OPLL (rs1464127693) [104]. However, another candidate gene association study of 162 Korean patients and 159 control subjects failed to reconfirm the association of some *BMP2* SNPs previously associated with OPLL, specifically rs2273073 and rs10490007 [78]. In addition to *BMP2*, there were a sib-pair linkage study and some candidate gene association studies reporting an association between BMP member (*BMP4* and *BMP9*) and OPLL (Table 2) [71–73, 104].

TGFβ1 protein is another key protein regulating bone and cartilage formation. Kamiya et al. firstly disclosed the possible association between a *TGFβ1* SNP (rs1800470) and OPLL in a candidate gene association study involving 46 Japanese patients and 273 control subjects [98], but this association was not confirmed by Han et al. in a candidate gene association study of 98 Korean patients and 200 control subjects [105]. Another candidate gene association study involving 369 Japanese patients and 258 control subjects also failed to associate the above-mentioned *TGFβ1* SNP (rs1800470) with OPLL, reporting instead that more than half of the patients harboring *TGFβ1* SNP (rs1800470) presented with OPLL that was not limited to cervical spine while most patients with a wild-type allele presented with OPLL limited to the cervical spine [106]. Therefore, this SNP might be a factor related to the area of the ossified lesion in the total spine in OPLL patients [106].

While a candidate gene association study selects a few genes as target genes for the analysis, a genome-wide association study (GWAS) comprehensively analyzes all genes, leading to the less biased information on the genetic factors associated with OPLL. The GWAS of OPLL involving 1130 Japanese patients and 7135 controls revealed SNPs in additional genes [75•]. While the odds ratios (ORs) for these SNPS with OPLL were not so high, SNPs in *CCDC91* (rs1979679, OR: 1.30), *HAO1* (rs2423294, OR: 1.43), and *RSPO2* (rs374810, OR: 1.35) were identified as increasing susceptibility. Additionally, increased gene expressions of *RSPH9* and *STK38L*, located within 1 Mb from rs927485 (OR: 1.37) and rs11045000 (OR: 1.32), respectively, were confirmed in osteoblasts, suggesting that these genes may be relevant for membranous ossification.

While the genetic background of OPLL is well established, the genetic factors associated with DISH are less so. A candidate gene association study in 97 Japanese DISH patients and 298 controls, and in 96 Czech DISH patients and 96 controls, revealed that a *COL6A1* SNP (rs35796750) already identified in OPLL was also associated with DISH in Japanese patients, although this association was undetected in Czech patients [76, 102]. Additionally, a recent study in Portugal utilized a mutational analysis via whole-exome sequencing in four patients with DISH to identify candidate pathogenic variants, followed by an association study of 65 patients and 118 control subjects, identifying SNP (rs34473844) in *PPP2RD* to be associated with DISH [103]. Moreover, because the clinical manifestation of DISH sometimes overlaps with OPLL, studies on the genetic factors of DISH were performed using cohorts of OPLL patients including cases who presented with imaging findings suggestive of DISH. For example, another candidate gene association study of *FGF2* and *FGFR* in 157 Korean OPLL patients including three patients with DISH and 222 controls reported that *FGF2* SNPs (rs1476217, rs3747676) were significantly associated with DISH [87].

### Heterozygote or Compound Heterozygote of the Mutation in Causative Genes Identified in the Monogenic Disorders Frequently Accompanied With the Spinal Ligament Ossification

**Fig. 1:**
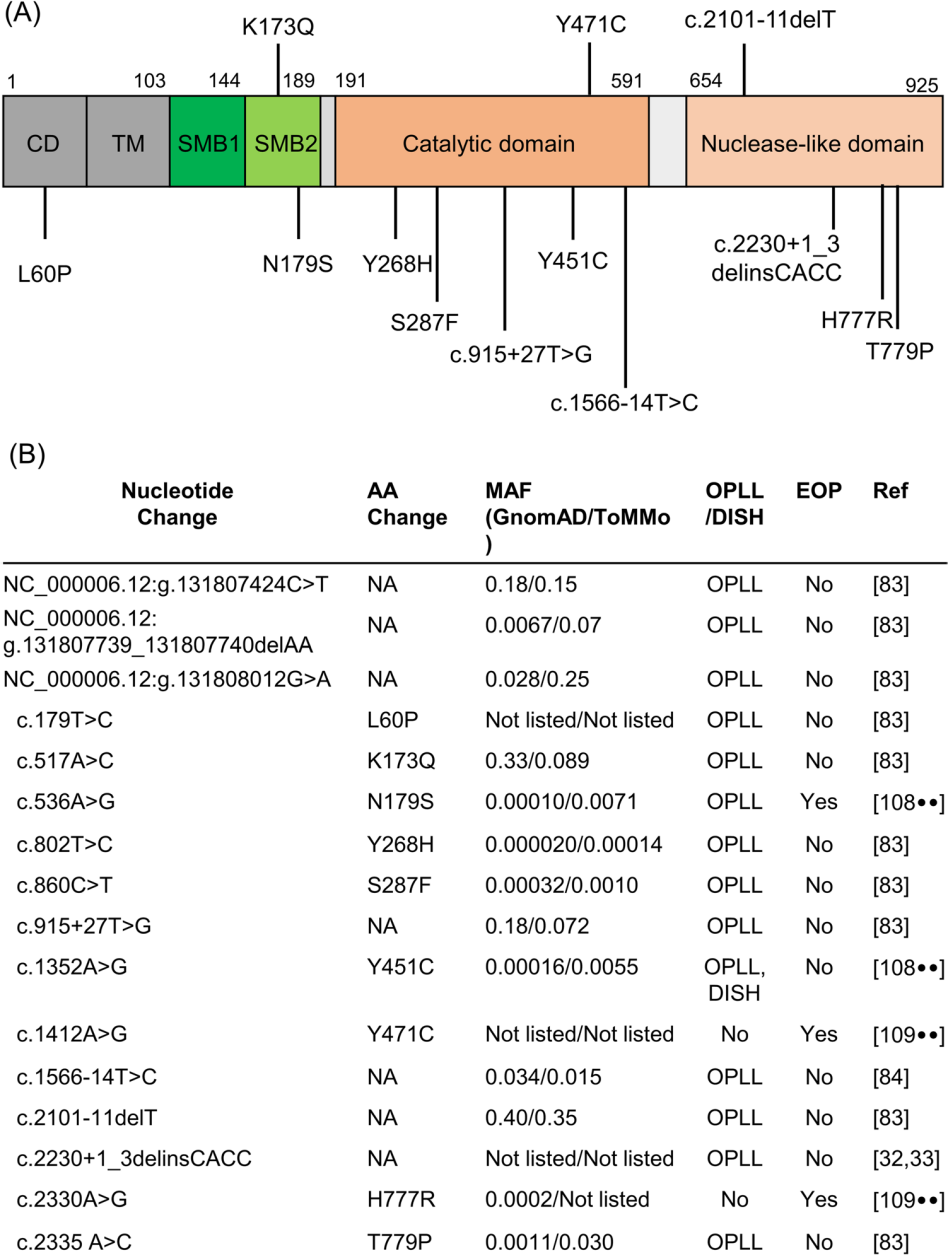
The location and allelic information of variants in ENPP1 gene detected in patients with OPLL. **A** The schematic image of ENPP1 protein and the location of each variant. **B** Bottom part: the list of allelic information and clinical phenotype of the ENPP1 variants detected in the patients with OPLL or osteoporosis. SMB, somatomedine B; AA, amino acid; MAF, minor allele frequency; OPLL, ossification of the posterior longitudinal ligament; DISH, diffuse idiopathic skeletal hyperostosis; EOP, early onset osteoporosis. Variant information is provided in accordance with GRCh38

After the identification of *ENPP1* as the causative gene in ttw mice with a severe OPLL phenotype, the same investigators searched for pathogenic *ENPP1* variants in 323 Japanese patients with OPLL, identifying 10 candidates (NC_000006.12:g.131808012G > A [rs1800949], NC_000006.12:g.131807739_131807740delAA [rs1799773], NC_000006.12:g.131808012G > A [rs1800948], c.179 T > C [rs1781304340], c.517A > C [rs1044498], c.802 T > C [rs17847050], c.860C > T [rs190947144], c.915 + 27 T > G [rs9493113], c.2101-11delT [rs397832689], c.2335A > C [rs1805138]) (Fig. 1) [83]. These pathogenic *ENPP1* variants occurred as monoallelic, biallelic, or compound heterozygous variants in OPLL patients, suggesting an association of *ENPP1* with the development of OPLL. The candidate variants were further assessed in an association study involving 332 control subjects, identifying only one variant (rs397832689) to be significantly associated with OPLL (Fig. 1) [83]. A second candidate gene association study involving 180 Japanese patients and 265 controls also reported a second SNP in *ENPP1* (c.1566-14 T > C [rs75272847]) to be associated with OPLL (Fig. 1) [84].

Recently, we described three patients with heterozygous or compound heterozygous *ENPP1* pathogenic variants (rs2273411 [GnomAD: 0.00010, ToMMo: 0.00071], rs201519006 [GnomAD: 0.00016, ToMMo: 0.0055]), who did not present with a GACI or ARHR2 clinical phenotype (Fig. 1). However, a patient with the a monoallelic *ENPP1* pathogenic variant (rs2273411) was found to have early onset male osteoporosis, and a second patient with a monoallelic *ENPP1* pathogenic variant (rs201519006) presented with DISH. Additionally, a third patient with compound biallelic *ENPP1* pathogenic variants (rs2273411/rs20159006) exhibited severe DISH/OPLL and enthesopathies in the Achilles’ tendon and around hip joints (Fig. 1) [107••]. In vitro analysis of the catalytic velocity in these ENPP1 variants confirmed decreased catalytic activity, a finding also supported by the decreased plasma PP_i_ levels observed in these patients (which is generated by the ENPP1 hydrolysis of ATP). This study also evaluated the presence of ectopic ossifications in family members of probands, identifying 23-year-old and 19-year-old male siblings harboring monoallelic *ENPP1* pathogenic variants who also possessed slight Achilles tendon enthesopathies but not OPLL. Interestingly, another study detected monoallelic pathogenic *ENPP1* variants previously identified in GACI patients with biallelic *ENPP1* deficiency (rs148462924 [GnomAD: 0.00011, ToMMo: not listed], rs147346173 [GnomAD: 0.00026, ToMMo: not listed]), but the monoallelic patients (60- and 69-year-old males) exhibited early-onset osteoporosis and multiple vertebral compression fractures. These findings of early onset osteoporosis and increased fracture risk were recapitulated in a mouse model of homozygous *ENPP1* deficiency (Fig. 1) [108••]. The combined data suggests that monoallelic *ENPP1* deficiency is a causative genetic factor inducing low bone mass.

The allelic frequency of previously reported pathogenic variants of *ENPP1* (rs2273411, rs20159006, rs148462924, rs147346173) detected in patients with OPLL or early-onset osteoporosis was as high as 0.5%. This frequency is much higher than the prevalence of monogenic disorders (i.e., XLH, ARHR1, and ARHR2), which are very rare (0.005%). In addition, the allele frequency of variants of susceptible genes identified by GWAS and association studies is about 30–40% (Table 2). Generally, the pathogenicity of the variants are inversely correlated with the allele frequency.

**Fig. 2:**
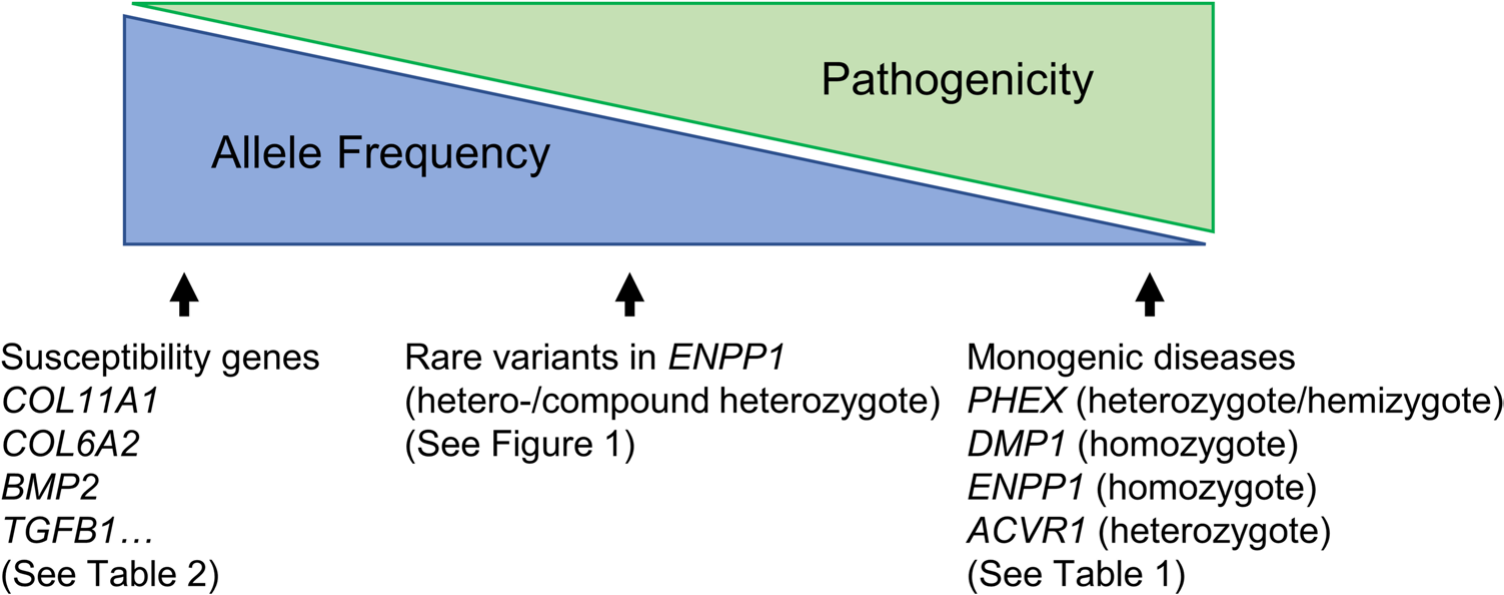
Schematic image of the clinical relevance to the spinal ligament ossification of susceptibility genes and genes responsible for monogenic diseases

Hence, the clinical impact of pathogenic *ENPP1* variants in monoallelic or compound biallelic patients on spinal ossification and spinal osteoporosis are midway between the more prevalent susceptible variants identified by GWAS or case–control studies (weak effect), and the less prevalent biallelic or compound monoallelic *ENPP1* and *DMP1* pathogenic variants in autosomal recessive GACI and ARHR1 patients (strong effect) (Fig. 2).

## Discussion

In this review, we focused on studies identifying genetic factors associated with spinal ligament ossifications. In monogenic diseases, the high frequencies of spinal ligament ossification occurring in XLH, ARHR1, and ARHR2 were reviewed. Hemizygous or heterozygous *PHEX* mutations and biallelic pathogenic variants of *ENPP1* and *DMP1* are strong genetic factors predisposing patients for spinal ligament ossification (Fig. 2). Biallelic *ENPP1* mutations, although rare, induce severe spinal ligament ossifications, whereas patients with monoallelic *ENPP1* pathogenic variants exhibit less severe spinal enthesopathies and have a reduced genetic risk of DISH and OPLL (Fig. 2). Finally, not all patients with monoallelic *ENPP1* deficiency exhibit spinal ligament ossification, suggesting that other susceptible variants in the variable genes detected by association studies (e.g., GWAS) and environmental factors (e.g., focal inflammation, diabetes mellitus, chronic trauma) may further modify the predisposition for spinal ligament ossifications [109].

Because *PHEX*, *DMP1*, and *ENPP1* all induce FGF23-related hypophosphatemia, they may affect common pathophysiological mechanisms inducing enthesopathies and spinal ligament ossifications. A Japanese study of OPLL patients reported that increased plasma FGF23 and lower plasma phosphate are associated with more rapidly progressing OPLL, additionally supporting FGF23 associated hypophosphatemia as a pathogenic mechanism for spinal enthesopathy [110, 111••]. Moreover, spinal ligament ossifications in patients with non-FGF23 mediated chronic hypophosphatemia are not increased. For example, patients with chronic FGF23-non-related hypophosphatemia (e.g., Fanconi syndrome, vitamin D deficiency) do not develop ectopic ossifications. Additionally, other forms of FGF23-related hypophosphatemia (e.g., tumor-induced osteomalacia or autosomal dominant hypophosphatemic rickets) are also not associated with spinal ligament ossification. We therefore hypothesized that unidentified mechanisms independent of serum phosphate and FGF23 in common with PHEX, DMP1, and ENPP1 may be responsible.

While the precise functions of PHEX and DMP1, even from the viewpoint of phosphate metabolism, remained to be elucidated, one definite function of ENPP1 is producing plasma PP_i_ — a potent inhibitor of ectopic calcification — through enzyme catalysis, and the regulating the bone mass via catalytic-independent pathways [112]. Plasma PP_i_ is markedly decreased in GACI and ARHR2 patients with biallelic *ENPP1* deficiency, and mildly reduced in the patients with monoallelic *ENPP1* deficiency [107••, 108••]. Moreover, plasma PP_i_ in the Hyp mouse, a murine model of XLH induced by *PHEX* mutations, is also reported to be decreased, supporting the notion that reduced plasma PP_i_ may induce the enthesopathies in XLH and ARHR2, resulting in spinal ossification and an OPLL phenotypes in these diseases [113]. A recent study demonstrated that recombinant ENPP1 normalized plasma PP_i_ and prevented enthesopathies in murine biallelic *ENPP1* deficiency, also supporting the hypothesis that plasma PP_i_ levels prevent enthesopathies [41•]. However, patients with homozygous inactivating mutations of *ABCC6* coding for the ABCC6 protein which promotes the cellular efflux of ATP may also develop GACI or pseudoxanthoma elasticum. These patients also exhibit low PP_i_ levels but not ectopic ossifications such as spinal ligament ossifications. However, because plasma PP_i_ in murine models of homozygous *ENPP1* and *ABCC6* deficiency are reduced to 10% and 30% of wild type, respectively, the difference in the prevalence of spinal ligament ossifications in these conditions may be related to the differences in plasma PP_i_ levels [108••, 114].

In addition to the genes associated with monogenic disorders and susceptible genes, acromegaly may be another relatively strong risk factor for spinal ligament ossification. Increased GH receptors in bone osteoblasts and insulin growth factor 1-induced osteogenic differentiation are proposed as the putative mechanisms for spinal ligament ossification in the patients with acromegaly [115, 116]. However, as with XLH, ARHR1, and ARHR2, not all acromegaly patients exhibit spinal ligament ossification [50]. Thus, acromegaly and the associated genes may be categorized as a relatively strong factors equivalent to heterozygous *ENPP1* deficiency.

Many case–control and GWAS studies have been conducted to identify OPLL susceptible genes, along with fewer studies focused on DISH, all mainly conducted in East Asia (Table 2). Because OPLL sometimes causes myelopathy due to spinal cord compression, patients with OPLL often visit hospitals and undergo surgery. As the clinical manifestations among some patients with OPLL, disease animal models and genetic studies (candidate gene association studies and GWAS) have been actively explored to elucidate the etiology of the disease and to develop treatment. On the other hand, regarding DISH, its prevalence is as high as 20–30% in the elderly [20, 21], and symptoms include impaired vertebral rotation and reduced bone mineral density in the vertebrae, sometimes leading to fractures. However, the affected paraspinal ligaments are mainly the anterior longitudinal ligaments among patients with DISH, and spinal cord compression is rarely observed, so there are fewer hospital visits and fewer opportunities for surgery compared to OPLL. As the clinical manifestations of DISH are mild, there might be less concern about exploring etiology and developing treatment, and the small number of patients visiting hospitals might also hinder a large-scale genetic study. Among the identified susceptible genes for OPLL, COL11A2 — the main collagenous component of cartilage — is speculated to play a protective role in ectopic ossification [80, 81, 117]. COL6A1 is a protein providing the structural support for osteoblasts or chondrocytes, and also plays a role in membranous or endochondral ossification [21]. Because *COL6A1* was identified as a susceptible gene in both OPLL and DISH, it may play a crucial role in the ossification process common to both disorders. BMP members, such as BMP2, BMP4, and BMP9, bind to receptors and activate several pathways, resulting in the transcription of proteins which promote proliferation and differentiation of mesenchymal stem cells, osteoblasts, and chondrocytes [21]. It is, therefore, highly probable that enhanced BMP signaling is associated with OPLL, a finding also supported by the identification of the p.K400E *ACVR1* activating variant which increases BMP signaling and is associated with DISH [42]. Among the identified susceptible genes for DISH, FGF2 transmits signals through FGFR2 and FGFR3, and FGF/FGFR is a major signal pathway involved in the bone formation [21].

Our laboratory identified that monoallelic and compound biallelic *ENPP1* pathogenic variants have moderate effects on spinal ligament ossification (Fig. 2). Therefore, ENPP1 enzyme replacement therapy may be a promising therapeutic strategy for treating OPLL/DISH in these patients. ENPP1-Fc enzyme replacement rescues murine models of GACI and ARHR2, including the low bone mass, lethal arterial calcifications, and the Achilles tendon enthesopathies observed in these models [41•, 118]. A clinical grade therapy is now in clinical development (INZ-701, Inozyme Pharma, Boston, MA) for patients with GACI, ARHR2, and PXE, and may be available to clinicians for other indications for the near future. Additionally, reference plasma PP_i_ levels were recently established in children and adolescents aged 0 to 18 years of age, enabling plasma PP_i_ as a predictive biomarker [119]. Measurement of plasma PP_i_, therefore, may efficiently screen patients with the spinal ligament ossifications for *ENPP1* deficiency.

In conclusion, related monogenic disorders of hypophosphatasia and acromegaly should be screened in the clinical assessment of patients with OPLL/DISH, and plasma PP_i_ is a new biomarker enabling clinicians to screen OPLL/DISH patients for *ENPP1* deficiency. Finally a therapeutic biologic ENPP1-Fc may be a promising therapy for patients suffering from spinal enthesopathies such as OPLL/DISH. The identification of common pathogenic mechanisms in monogenic and endocrine disorders associated with OPLL/DISH, and the development of targeting therapeutics for other relevant pathways such as ACVR1, COL11A2, COL6A, BMP2,4,9, and TGF-β1 are remaining important questions to be addressed.

## Data Availability

The datasets generated in the current review article are not publicly available but are available from the corresponding author on reasonable request.
